# On the dependence of creep-induced dislocation configurations on crystallographic orientation in pure Al and Al–Mg

**DOI:** 10.1107/S1600576723003771

**Published:** 2023-05-29

**Authors:** Ricardo Fernández, Gizo Bokuchava, Giovanni Bruno, Itziar Serrano-Muñoz, Gaspar González-Doncel

**Affiliations:** a Centro Nacional de Investigaciones Metalúrgicas (CENIM), Consejo Superior de Investigaciones Científicas, Avenida de Gregorio del Amo 8, E- 28040 Madrid, Spain; b Frank Laboratory of Neutron Physics, Joint Institute for Nuclear Research, Joliot-Curie Street 6, 141980 Dubna, Moscow Region, Russian Federation; c Bundesanstalt für Materialforschung und -prüfung (BAM), Unter den Eichen 87, D-12200 Berlin, Germany; d Institute of Physics and Astronomy, University of Potsdam, Karl-Liebknecht-Straße 24–25, D-14476 Potsdam, Germany; SLAC National Accelerator Laboratory, Menlo Park, USA

**Keywords:** creep, aluminium alloys, dislocations, fractals, diffraction peak width

## Abstract

The peak broadening in neutron diffraction experiments on tensile specimens of pure Al (99.8%) and an Al–Mg alloy pre-deformed at different creep strains is analysed. These results are combined with the kernel angular misorientation of electron backscatter diffraction data from the creep-deformed microstructures. It is found that differently oriented grains possess different microstrains. These microstrains vary with creep strain in pure Al, but not in the Al–Mg alloy. It is proposed that this behaviour can explain the power-law breakdown in pure Al and the large creep strain observed in Al–Mg. The present findings further corroborate a description of the creep-induced dislocation structure as a fractal, predicated on previous work.

## Abstract 2

This work describes the influence of crystallographic orientation on dislocation arrangements during primary and secondary creep of polycrystalline Al and Al–Mg.

## 1. Introduction

The creep behaviour of aluminium alloys processed by ingot metallurgy has been extensively investigated at the microscale (Kassner, 2020 ▸, 2004 ▸; Jiang *et al.*, 2013 ▸; Lin *et al.*, 2011 ▸; Tello *et al.*, 2010 ▸; Fernández & González-Doncel, 2007 ▸; Gariboldi & Casaro, 2007 ▸; Burt & Wilshire, 2006 ▸; Sato *et al.*, 1997 ▸). In the classical microscopic description, high-temperature deformation occurs by dislocation motion, the dynamics of which are basically controlled by atomic self-diffusion. The specific dislocation/lattice defect interactions and their dependence with temperature confer, in essence, the creep behaviour of these alloys (Sherby & Burke, 1968 ▸). In this context, the grain/subgrain effect on this behaviour is only relevant for very small sizes (Sherby & Burke, 1968 ▸).

In a recent set of papers, a new description of the creep behaviour of aluminium alloys has been proposed (Fernández *et al.*, 2016 ▸, 2018*a* ▸). In particular, a new solid-state transformation creep (SSTC) model (Fernández *et al.*, 2016 ▸) has been developed. This model provides a completely different perspective on creep in aluminium alloys. The description is developed at the mesoscale, and thereby at the scale of the material’s microstructure, *i.e.* above that of the grain size.

One of the main ideas considered in the SSTC model is that the dislocation population evolves during creep deformation from an initial random arrangement up to a defined structure formed in the secondary creep stage (minimum strain rate). These structures present a fractal arrangement, which increases the pipe diffusion contribution in the system and determines the evolution of strain rate during creep.

In spite of the proofs provided by Fernández *et al.* (2016 ▸, 2018*a* ▸) and Cabeza *et al.* (2018 ▸), the above idea that fractal-like dislocation structures are generated in aluminium alloys during creep strain requires further experimental confirmation. The fractal nature of the dislocation structures in creep-deformed metals is thought to be a key factor that could elucidate some of the still unexplained points in relation to creep in metallic alloys, such as creep power-law breakdown and the wild variation in the pre-exponential term reported in the literature (Fernández *et al.*, 2020*a* ▸).

In our model, we assume that these structures influence the creep behaviour by modifying the effective diffusion. The fractal nature of dislocation structures developed during creep is considered in the SSTC model, together with other microstructural characteristics such as the grain size, the mobile/total dislocation densities, the Burgers vector, the equilibrium vacancy concentration around dislocations, and the diffusion coefficient of such vacancies (Fernández *et al.*, 2020*a* ▸). These dislocation arrangements are very difficult to characterize by transmission electron microscopy (TEM) in a statistically relevant manner, due to their multiscale fractal structure (Cabeza *et al.*, 2018 ▸; Fernández *et al.*, 2020*a* ▸,*b* ▸) and the limited field of view of TEM. Complementary studies at the mesoscale (100–1000 µm) have been conducted using X-ray refraction radiography (Cabeza *et al.*, 2018 ▸). Some evidence of subgrain formation and damage progress was provided, but no crystallographic information was obtained, since X-ray refraction is not sensitive to crystal orientation. *In situ* synchrotron tests have provided some advances in the understanding of the creep deformation (Al Mamun *et al.*, 2021 ▸).

In this context, electron backscatter diffraction (EBSD) is commonly used when examining microstructural evolution at the micro- and mesoscale, including the effect of the crystal structure and orientation, and even the distribution of dis­locations (Carneiro & Simões, 2020 ▸; Wilkinson & Britton, 2012 ▸; Humphreys, 2001 ▸). In EBSD analysis, the kernel average misorientation (KAM), defined as the average misorientation angle of a given point in a map with respect to all neighbouring points, is typically applied to evaluate the spatial distribution of plastic deformation (Wright *et al.*, 2011 ▸). The KAM is also commonly considered as a qualitative description of the dislocation density distribution: an increase in the dislocation density leads to increasing misorientation angles (Muránsky *et al.*, 2019 ▸; Rui *et al.*, 2019 ▸). Using EBSD+KAM, we have recently observed in creep-deformed Al-99.8% samples that different dislocation arrangements are formed as a function of crystal orientation; we have assigned the origin of this phenomenon to the influence of intergranular stress developed during the extrusion process (Serrano-Munoz *et al.*, 2022 ▸). In spite of this previous work, the grain crystallographic orientation has so far been barely considered in creep models (Fernández *et al.*, 2020*a* ▸) (including SSTC), while one would expect that this should greatly influence the creep behaviour of metals (Wang *et al.*, 2022 ▸).

In addition to EBSD, the dislocation density can be determined as a function of crystal orientation by means of diffraction techniques using highly penetrating radiation, such as neutrons and/or synchrotron X-rays. The capability of diffraction techniques to detect microstructural features (and changes) is well proven because diffraction data contain enough information to be statistically representative.

It has been previously demonstrated, using neutron time-of-flight (TOF) diffraction, that plastic deformation induces broadening of the individual diffraction peaks (Bokuchava, 2016 ▸). Average microstrains within the grains could be determined by Ungár’s model (Ungár *et al.*, 1999 ▸). The square of the peak broadening is calculated as

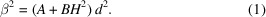


*d* is the lattice spacing, *A* and *B* are fitting parameters, and *H* ^2^ is the anisotropy factor (for cubic materials),

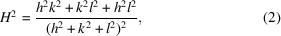

where *h*, *k* and *l* are the Miller indices.

Typically, TOF diffractometers operate over a rather wide range of interplanar spacings *d* and consequently allow simultaneous recording of a large number of diffraction peaks. For a TOF diffractometer, the width of a diffraction peak Δ*d* as a function of *d* is given by (Bokuchava, 2016 ▸)

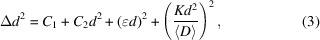

where *C*_1_ and *C*_2_ are the time and geometric components of the instrument resolution function, respectively, and their values are found from measurements with a standard sample. The parameter ɛ = (Δ*a*/*a*) is the variance of the unit-cell parameter (the microstrain), 〈*D*〉 is the size of coherently scattering domains (crystallites) and *K* is the Scherrer constant (∼0.94), which takes into account the shape of the crystallites.

The dependence Δ*d* ^2^(*d* ^2^) is linear, with a high slope compared with a standard sample, when the peak broadening is caused only by the presence of microstrain and the effect of the finite crystallite size is negligible (large crystallites). In the case of small crystallites (typically <100 nm), peak broadening due to the grain size effect is pronounced and gives a characteristic parabolic dependence. Usually, it is convenient to subtract the instrument resolution function [see equation (1)] and then analyse the diffraction peak broadening

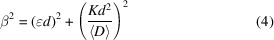

related only to the material properties using the Williamson–Hall method (Williamson & Hall, 1953 ▸). Accordingly, by plotting the β^2^(*d* ^2^) dependences over a sufficiently large range of *d* values, one can easily separate the contributions to the diffraction peak broadening, *i.e.* those due to microstrain ɛ and finite crystallite size 〈*D*〉, and determine these microstructural parameters separately.

Equation (1) can be used to estimate the ratio between edge and screw dislocations in a material (Hajyakbary *et al.*, 2015 ▸). The dislocation content can be studied as a function of the crystal orientation by following the behaviour of the broadening of different diffraction peaks as a function of increasing creep deformation.

Stored dislocations that are necessary to maintain the strain compatibility (by accommodating the lattice curvature resulting from long-range deformation gradients) are commonly known as geometrically necessary dislocations (GNDs) (Nye, 1953 ▸; Ashby, 1970 ▸; Hughes *et al.*, 2003 ▸). The net geometric effect of GNDs is generally considered by a net non-zero Burgers vector. On the other hand, statistically stored dislocations (SSDs, such as dislocation forests) refer to those that accommodate homogeneous deformations via compensating statistical trapping processes (*e.g.* by forming dipoles or tangles). SSDs have no net cumulative effect in the lattice curvature and therefore should not be detectable from the lattice curvature. As such, EBSD can only detect GNDs. The total dislocation density in a polycrystalline aggregate is the sum of GND and SSD densities (Nye, 1953 ▸), and both dislocation types play a role in the plastic deformation process.

The distinction between GNDs and SSDs can be considered as a length-scale problem: GNDs transition into SSDs (*i.e.* more dislocations cancel each other) with increasing length scale (Muránsky *et al.*, 2019 ▸; Kysar *et al.*, 2010 ▸). On the other hand, diffraction line profile analyses of reverse time-of-flight (RToF) diffraction patterns are by default averaged over many grains, conveying information about the total amount, ρ_Tot_, of all stored dislocations (*i.e.* the sum of GNDs and SSDs). Typically, the GND density is controlled by microstructural characteristics such as grain size and texture, while the density of stored SSDs is dependent on the material but somewhat unrelated to the characteristics of the microstructure.

The two materials studied here, Al-99.8% and Al–3.85%Mg alloy, possess very different creep behaviours. In the case of Al-99.8%, a subgrain structure is formed during creep. In the case of Al–Mg alloys, dislocation forests (structures of homogeneously distributed dislocations) are formed. In this context, the aim of the present work is to investigate the correlation between the dislocation substructures generated during creep and both the intergranular strain and the crystal orientation inherited from the extrusion process. For this purpose, we used neutron diffraction to investigate strains, and both neutron diffraction and EBSD to investigate the effect of crystal orientation on the dislocation arrangements.

## 2. Materials and methods

The samples used in the present investigation were machined from hot extruded bars of Al-99.8% and Al–3.85%Mg alloy. X-ray diffraction texture measurements were performed by means of the Schulz reflection method on a Siemens D5000 diffractometer equipped with a Cu anode (Cu *K*α radiation) and an Euler cradle for the rotations.

The creep tests were conducted at constant stress thanks to an Andrade cam available on the creep machines. Cylindrical tensile samples were machined with the tensile axis parallel to the extrusion direction, with 10 mm gauge length, 3 mm diameter and threaded heads for the experiment. The elongation was recorded as a function of time by two digital strain gauges (Solartron model DP/5/S) with a sensitivity of 0.2 µm. The applied load was monitored with a Transdutec load cell (model TSC-1/2500N). The clamping system allowed suppression of the contributions of the machine and the grip compliance to the total strain. The tests were conducted at 573 K and two stress levels, 21 and 29 MPa. These stresses belong to the stress interval ranging from the power-law creep to the power-law breakdown regime. A heating rate of 100 K h^−1^ was used from room temperature to 573 K, followed by a 1 h soaking time. The samples were deformed up to 1, 2 and 3% strain for Al-99.8% and 1, 3 and 6% strain for Al–3.85%Mg. In order to ‘freeze’ the dislocation arrangements corresponding to each strain level, the samples were quickly cooled using compressed air under the action of the applied stress (21 or 29 MPa) from 573 K to room temperature. This process took around 3 min, *i.e.* a cooling rate of around 6000 K h^−1^ was achieved (therefore much higher than the heating rate of 100 K h^−1^). These samples were used for both the *ex situ* neutron diffraction measurements and the EBSD–KAM analysis.

Neutron diffraction spectra were acquired with counting times of about 12 h per spectrum. All neutron diffraction experiments were performed on the Fourier stress diffractometer (FSD) at the IBR-2 pulsed reactor at FLNP JINR (Dubna, Russia) (Bokuchava, 2018 ▸). The cylindrical samples were mounted in the vertical orientation, and the gauge volume used was 0.1 cm^3^ with a large number of grains (∼10^5^) in the neutron beam. In the horizontal sample mount (*i.e.* axial component measurement) the irradiated gauge volume would have been elongated, and such an arrangement would have given some additional contribution to the geometric component of the resolution function. Since such a contribution is hard to estimate, only the radial strain component was investigated. It must be noted that the vertical sample mount corresponds to the FSD reference sample measurement (standard sample in vertical position). Such a configuration gives no extra broadening due to geometric effects.

**Figure 1:**
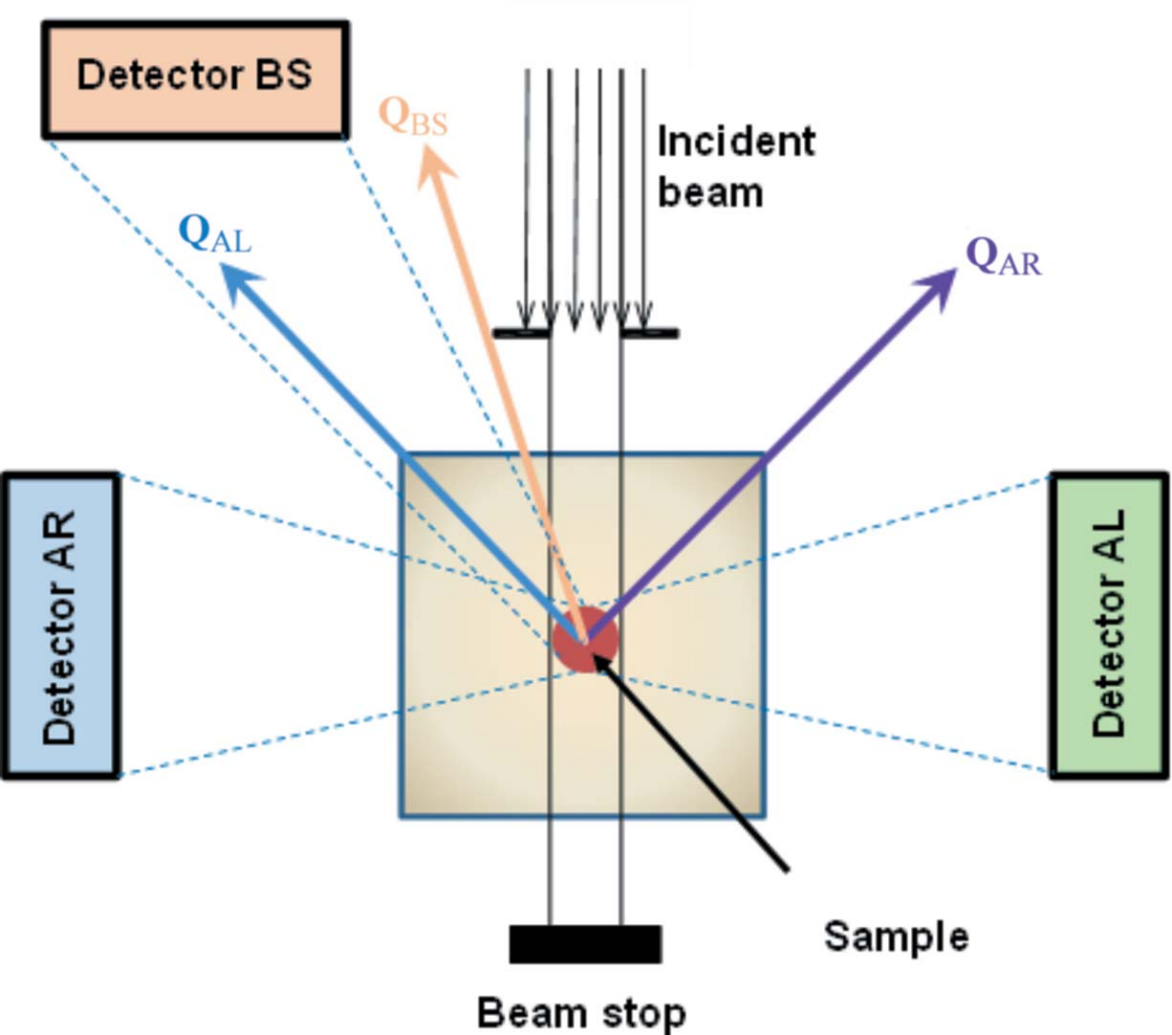
A diagram of the experiment on the FSD neutron diffractometer. The BS detector was used for the present investigation.

A special correlation technique at the long-pulse neutron source was used on the FSD, *i.e.* a combination of the fast Fourier chopper for the primary neutron beam intensity modulation and the RToF method for data acquisition. This allowed us to obtain a high resolution [Δ*d*/*d* ≃ 2 × 10^−3^ for the backscattering (BS) detector used in the present work and Δ*d*/*d* ≃ 4 × 10^−3^ for the ±90° detectors at *d* = 2 Å] over a wide range of interplanar spacing *d_hkl_* while using a relatively short flight distance between the chopper and the sample position (*L* = 5.55 m). A sketch of the FSD instrument configuration is shown in Fig. 1 ▸.

All main diffraction peaks from the Al phase were indexed with a face-centred cubic (f.c.c.) structure (space group *Fm*3*m*) and a lattice parameter *a* ≃ 4.050 Å. The measured diffraction spectra were processed by the *MRIA* program (Zlokazov & Chernyshev, 1992 ▸) for full profile analysis based on the Rietveld method (Rietveld, 1969 ▸). A special procedure implemented in *MRIA* (similar to the Williamson–Hall peak broadening analysis; Williamson & Hall, 1953 ▸) was used to calculate microstrains from RToF data. Al-99.8% and Al–Mg powders with particle sizes below 100 µm were used as unstrained references.

The EBSD–KAM measurements were made under the same conditions as used by Serrano-Munoz *et al.* (2022 ▸, 2023 ▸). The reader is referred to these references for further details about sample preparation, parameters used for data collection, and the procedure for data processing and evaluation. In the current work there are two main changes to the data treatment for better comparison with the neutron diffraction results: (i) the EBSD orientation maps were taken on a sample section parallel to the radial axis (instead of the loading axial axis), and (ii) the KAM maps were fitted to the range between 0 and 2° for better comparison between the two investigated materials.

## 3. Results and analysis

### 3.1. Microstructure

**Figure 2:**
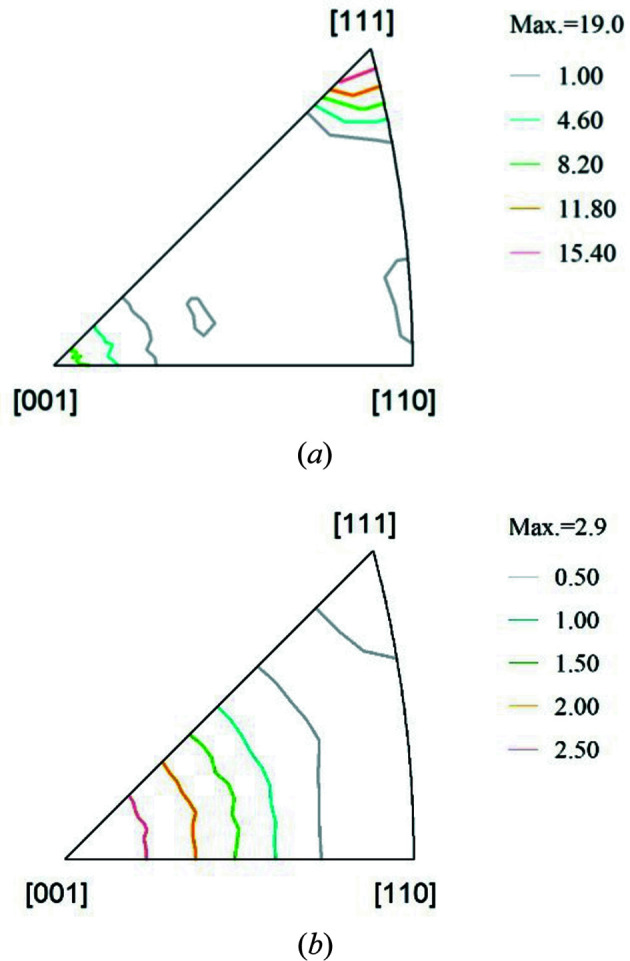
XRD texture, in the form of inverse pole figures for the extrusion axis direction, for (*a*) Al-99.8% and (*b*) Al–3.85%Mg alloy.

The materials investigated present different textures, despite the fact that both were extruded under similar temperature and pressure conditions. This result is an indication that the addition of Mg atoms into the Al lattice generates a very strong microstructural effect. In Fig. 2 ▸ the texture of both materials is shown in the form of inverse pole figures for the extrusion axis direction. Al-99.8% shows the typical 〈111〉+〈100〉 fibre texture (with the fibre axis parallel to the extrusion direction) of extruded f.c.c. metals, while Al–3.85%Mg shows a 〈100〉+〈111〉 fibre texture. The volume fractions of different grain families with specific 〈*hkl*〉 oriented along the axial direction were calculated by integrating the orientation distribution function in a 15° solid angle around each crystallographic direction using the *TexTools* software (Resmat, Montreal, Canada) (Table 1 ▸). In Al-99.8%, 45% of grains have their 〈111〉 axis aligned along the axial direction. In the Al–3.85% alloy, 50% of grains have their 〈100〉 axis aligned along the axial direction. The grain size of both materials is around 100 µm.

### 3.2. Creep tests

**Figure 3:**
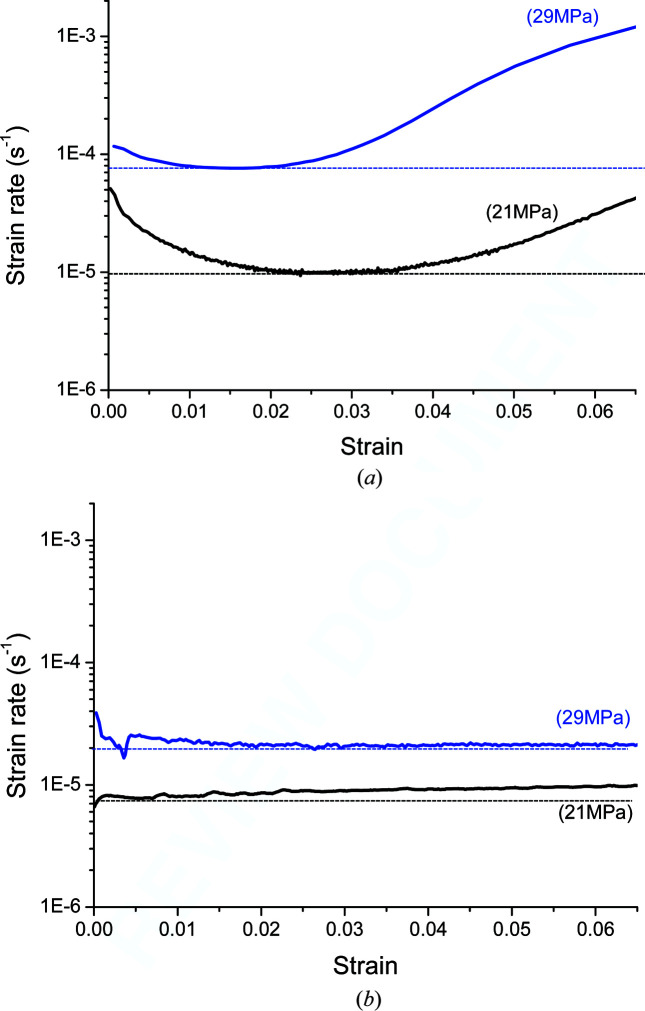
Strain rate as a function of creep strain at 573 K for (*a*) Al-99.8% and (*b*) Al–Mg alloy.

Creep curves (in the form of strain rate versus strain) are shown in Fig. 3 ▸. The dashed lines indicate the minimum strain rate. The creep strength of the Al–3.85%Mg alloy is higher than that of Al-99.8% (Table 2 ▸). The strain rate depends on the applied stress (and, as shown below, on the dislocation structure). The creep behaviour of the two materials is remarkably different even though they underwent the same heat treatment history and/or mechanical processing procedure (Fig. 3 ▸).

### 3.3. Neutron diffraction: microstrain evolution with creep deformation

**Figure 4:**
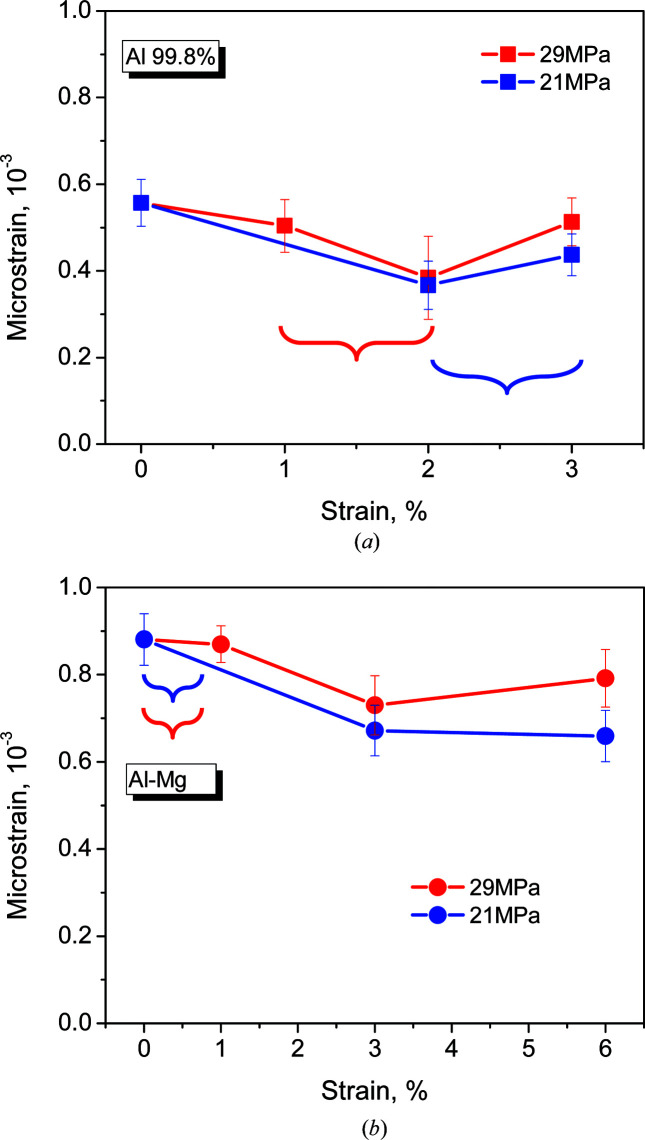
Microstrains as a function of creep strain for (*a*) Al-99.8% and (*b*) Al–3.85%Mg.

*Undeformed samples.* No macroscopic (Type I) residual stress (RS) was expected after the heat treatment at 573 K prior to the creep tests (Fernández *et al.*, 2018*b* ▸). However, intergranular (Type II) and intragranular (Type III) microstresses are typically generated in the extrusion process and may remain after the heat treatment (Serrano-Munoz *et al.*, 2022 ▸). Type III microstrains were determined by Δ*d* ^2^ versus *d* ^2^ (Williamson–Hall) plots, once the linear instrument resolution function had been subtracted. (Note that a certain contribution of Type II stresses cannot be discarded, so in the following we call such strains simply ‘microstrains’.) The remaining pure peak broadening dependences (their slopes) give an estimation of average microstrains in the probed volume (Fig. 4 ▸). Both Al-99.8% and the Al–3.85%Mg alloy present non-zero microstrains. Such strains are related to the extrusion process (*i.e.* they are already present in the 0% strain samples). The analysis shows higher microstrains for the Al–3.85%Mg alloy. As for the texture intensity, the higher Type III microstrains are related to the Mg solid solution.

*Deformed samples.* The minimum strain rate regions for Al-99.8% at 21 and 29 MPa are indicated by braces in Fig. 4 ▸(*a*). Microstrains vary in the range roughly 350–550 µɛ for Al-99.8% and roughly 650–900 µɛ for Al–3.85%Mg. The average microstrain hardly varies for either Al-99.8% or Al–Mg during creep deformation, independent of the applied stress, considering an error bar of about ±100 µɛ (Fig. 4 ▸). As mentioned above, during creep deformation Al–Mg always possesses the largest microstrains. Interestingly, a decrease in the microstrain is observed at 2% creep strain in Al-99.8% and at 3% in Al–Mg.

The dislocation density ρ can be estimated from microstrain values according to a simple model proposed by Williamson & Smallman (1956 ▸),

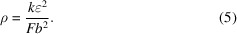

ɛ is the average microstrain, *b* is the magnitude of the Burgers vector, *F* is a parameter describing the interaction of dislocations (in the present case, *F* = 1) and *k* = 12*A*, where *A* = 2 corresponds to a Lorentzian peak shape and *A* = π/2 to a Gaussian shape. With the application of equation (5), it is assumed that both GNDs and SSDs contribute to the diffraction peak broadening.

**Figure 5:**
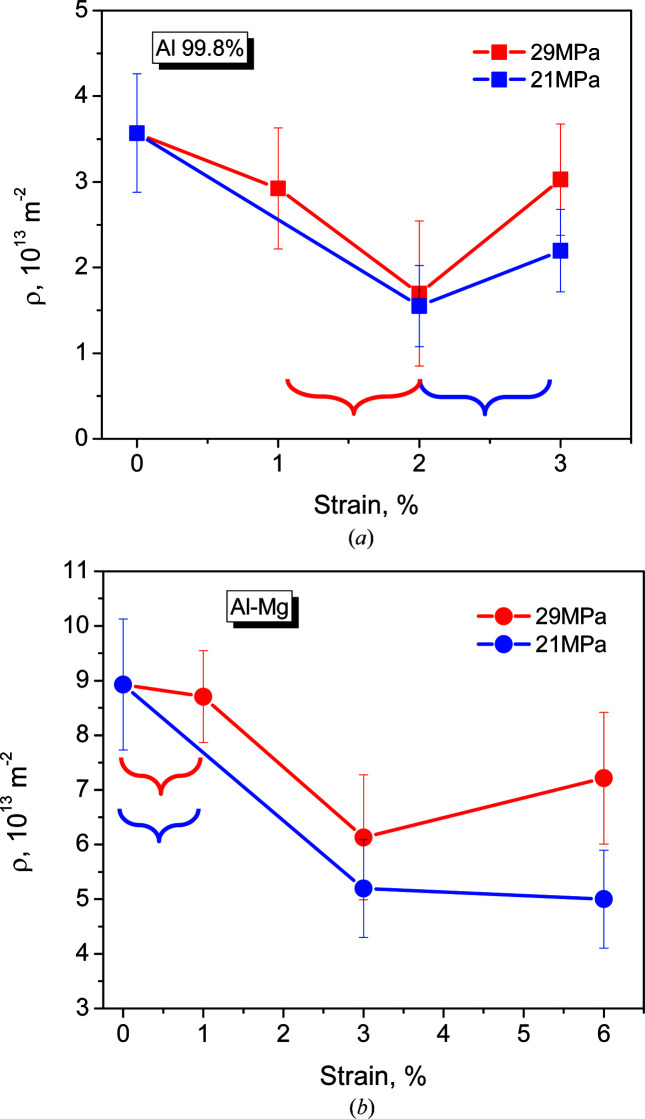
Evolution of dislocation density during creep with increasing creep strain for (*a*) Al and (*b*) Al–3.85%Mg alloy. Curly brackets indicate the secondary creep stage.

The dislocation density decreases to about half of the initial value when the creep strain reaches 2% for Al-99.8% [Fig. 5 ▸(*a*)] and 3% for Al–3.85%Mg [Fig. 5 ▸(*b*)]. This mirrors the microstrain decrease mentioned above. The evolution of the dislocation density with creep strain is practically independent of the applied stress in Al-99.8% considering the error bars [Fig. 5 ▸(*a*)]. In the case of Al–3.85%Mg, the dis­location density is around 30% higher at 29 MPa [Fig. 5 ▸(*b*)], but the evolution of the dislocation density with creep strain is similar for the two applied stresses. This shows that the capacity for dislocation rearrangement during creep of Al–Mg, linked to the microstrain, is inversely proportional to the applied stress, *i.e.* to the drag effect introduced by Mg atoms anchored to dislocations. This drag effect, limiting dislocation rearrangement, grows with the dislocation density. Finally, for creep strains above 2% in Al-99.8% and 3% in Al–3.85%Mg, there is an increase in the dislocation density with respect to its minimum.

**Figure 6:**
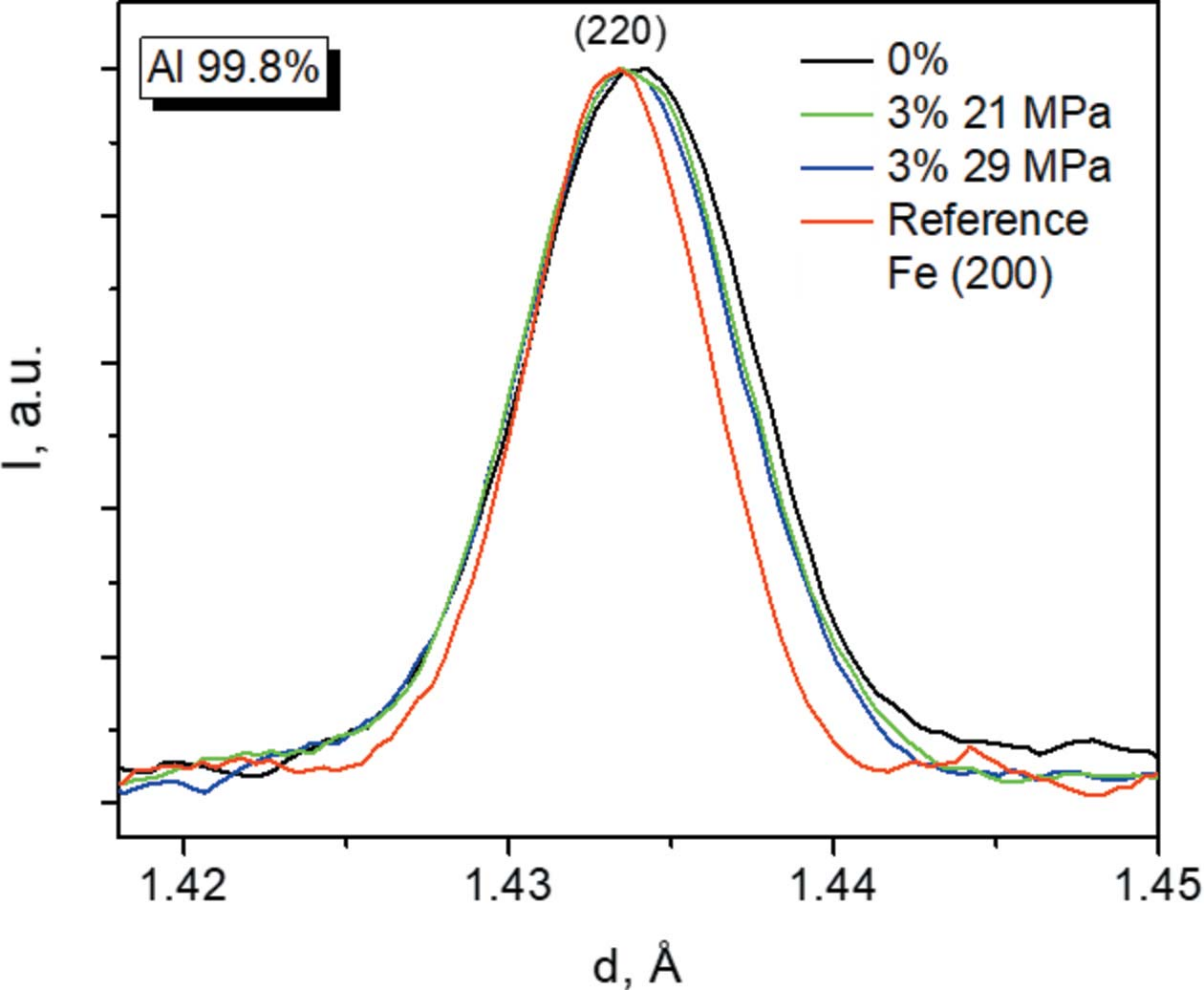
Broadening of the neutron diffraction peak Al-220 during creep for the Al-99.8% sample series. The 200 peak of a reference α-Fe powder is also shown.

**Figure 7:**
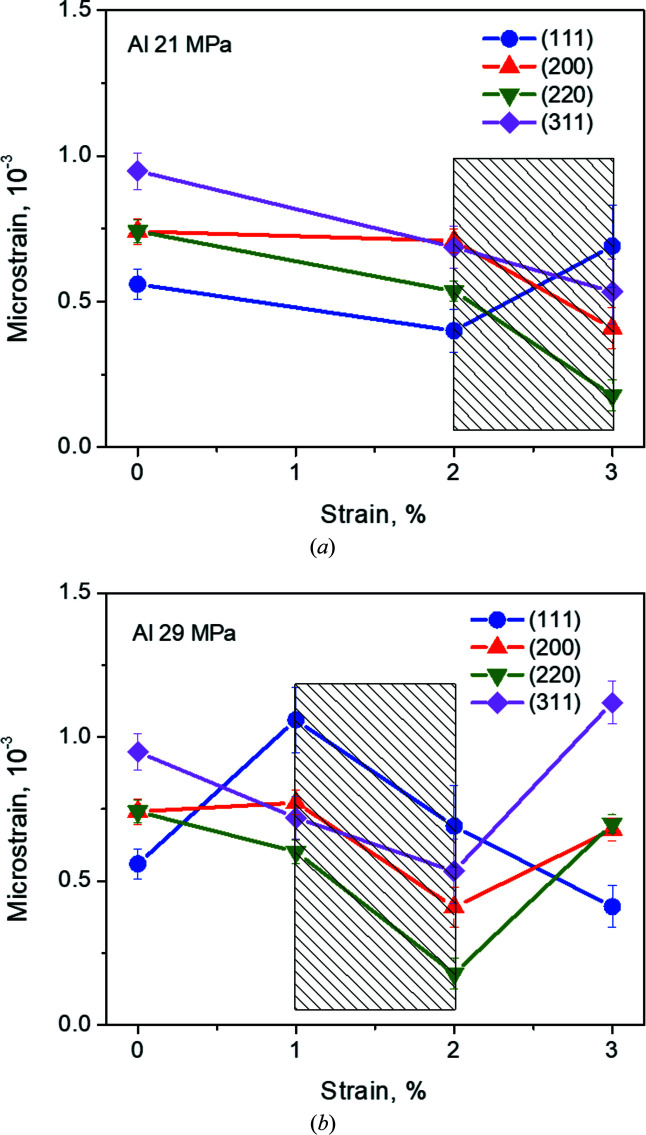
Microstrain evolution during creep of grains with different crystallographic orientations for Al-99.8%, (*a*) at 21 MPa and (*b*) at 29 MPa. Hatched rectangles indicate the secondary creep stage.

Hitherto, we have only studied the average microstructural creep evolution of the two materials (Figs. 4 ▸ and 5 ▸). In what follows, we also exploit the ability of diffraction to separate the contribution of the different grain families. The evolution of the 200 peak of Al-99.8% after 0 to 3% creep strain is shown in Fig. 6 ▸. Figs. 7 ▸ and 8 ▸ show the microstrain evolution with creep strain for different grain families for Al-99.8% and Al–3.85%Mg, respectively. In the case of Al-99.8%, the initial microstrain level (at 0% creep strain) is the highest for the {311} family, decreasing along the sequence {311} → {220}/{200} → {111} (Fig. 7 ▸). Note that the difference in microstrain among grain families has been associated with the presence of intergranular (Type II) stresses inherited from the extrusion process (Serrano-Munoz *et al.*, 2022 ▸; Fernández *et al.*, 2018*b* ▸). For all families except for the {111} grains there is a microstrain decrease as a function of creep strain, until the minimum strain rate is reached. This behaviour is independent of the applied stress. The only difference is that a steady state is reached at 1% strain at 29 MPa and at 2% strain at 21 MPa. However, the microstrain evolution of {111} grains with creep strain greatly depends on the applied stress. At 21 MPa, the microstrain of {111} grains diminishes in the primary state, reaching a minimum at the beginning of the creep steady state and showing a strong increase during the steady state [Fig. 7 ▸(*a*)]. Since microstrain and dislocation density are proportional to each other, this trend reproduces the one shown by the dislocation density in Al-99.8% during the creep test. However, at 29 MPa, the {111} microstrain evolution with creep strain is opposite to that at 21 MPa [Fig. 7 ▸(*b*)]. Interestingly, the trend shown by the evolution of dislocation density during the creep test at 29 MPa is followed by all the components except for {111}.

**Figure 8:**
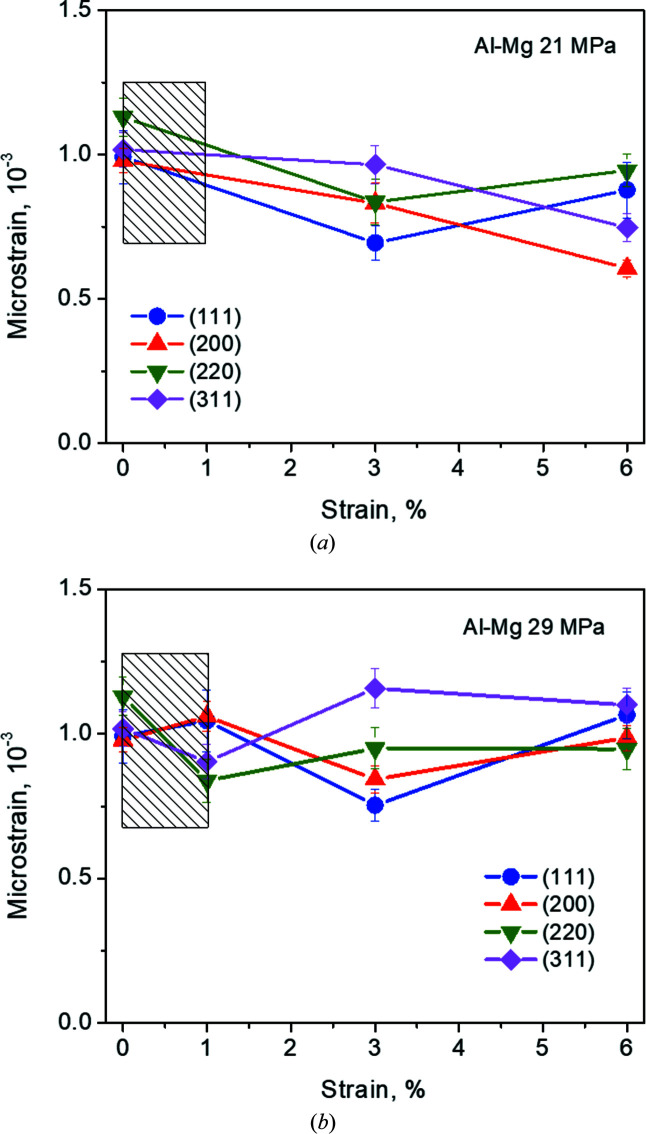
Microstrain evolution with increasing creep strain of grains with different crystallographic orientations for Al–3.85%Mg, (*a*) at 21 MPa and (*b*) at 29 MPa. Hatched rectangles indicate the secondary creep stage.

In the case of Al–3.85%Mg (Fig. 8 ▸) the initial microstrain level (at 0% creep strain) is very similar for all the components investigated. However, their evolution with creep strain at 21 MPa [Fig. 8 ▸(*a*)] can be divided into two groups. On one hand, the {200}/{311} microstrains decrease with creep strain up to 6% strain. On the other hand, the {220}/{111} microstrains first decrease in the primary regime (ɛ < 3%) and then increase in the steady state.

At 29 MPa [Fig. 8 ▸(*b*)] the microstrains show small variations with creep strain. The {220}/{311} grains and {200}/{111} grains behave in opposite ways. If the microstrains in one group decrease, they increase in the other group and *vice versa* up to ɛ = 6%.

**Figure 9:**
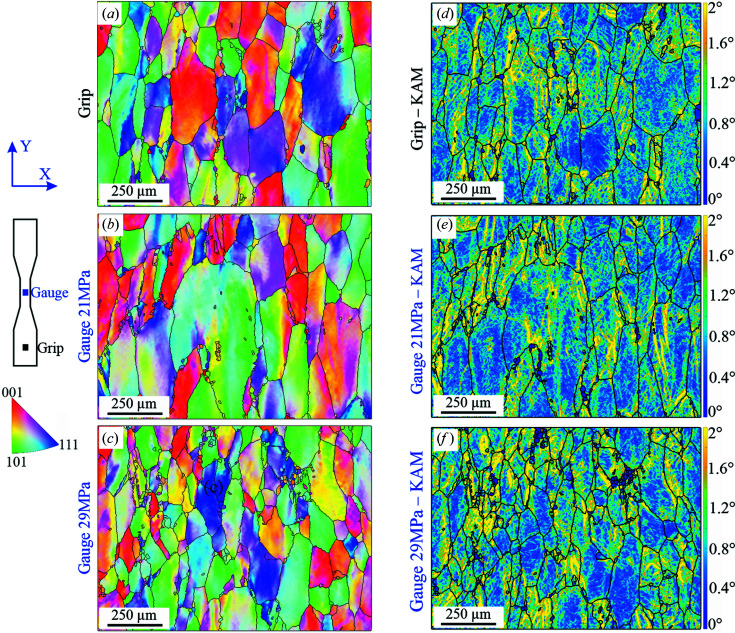
EBSD orientation maps of Al-99.8%. (*a*) Undeformed condition (grip region). (*b*) Creep deformed at 2.5% strain and 21 MPa. (*c*) Creep deformed at 2.5% strain and 29 MPa. The corresponding KAM angle maps are shown in panels (*d*), (*e*) and (*f*), respectively. The IPF colour reference triangle is for the radial direction (*X*).

Using EBSD maps at ×250 magnification of the Al-99.8% material (Fig. 9 ▸) we can observe that the accumulation of strain is very heterogeneous within individual grains. Grains show a combination of low misorientation areas (<0.5°, blue) and regions where the misorientation saturates to >2° (yellow). When comparing different grains, there is also considerable heterogeneity of the misorientation spatial arrangement. However, no significant differences are observed among the different conditions [Figs. 9 ▸(*d*)–9 ▸(*f*)] or crystal orientations. It is commonly accepted that pure metals, such as Al-99.8%, accumulate strain mainly in the form of GNDs; this implies that most of the strain accumulation occurring in this material can be detected by means of EBSD (Muránsky *et al.*, 2019 ▸).

**Figure 10:**
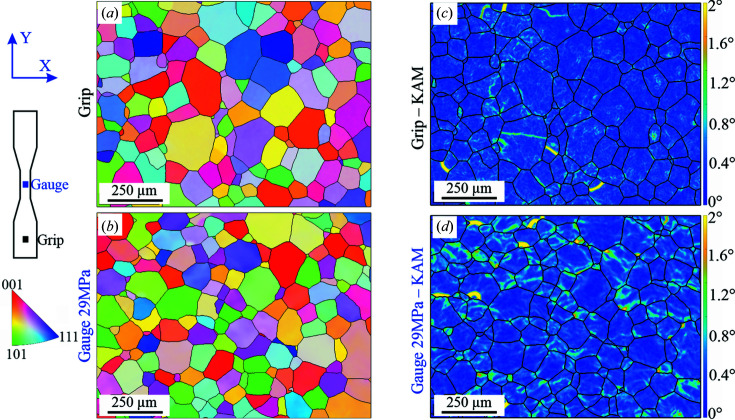
EBSD orientation maps for Al–3.85%Mg. (*a*) Undeformed condition (grip region). (*b*) Creep deformed at 2.5% strain and 29 MPa. The corresponding KAM maps are shown in panels (*c*) and (*d*), respectively. The colour reference triangle is for the radial direction (*X*).

In contrast, Al–3.85%Mg accumulates strain in the form of dislocation forests (SDD), which are expected to have very little influence on intragranular microstrain generation (the lattice curvature mentioned above), as is seen by comparing Figs. 7 ▸ and 8 ▸. Therefore, the KAM results given in Fig. 10 ▸ show misorientation levels close to 0° in wide areas of the maps. Nevertheless, some regions with higher misorientations can be observed in the form of bands. In addition, differences between conditions can be captured at ×250 magnification. In the case of the grip (0% strain), the strain accumulation occurs in the form of vermicular lines, which exhibit an average misorientation of about 1°. Note that the heterogeneity of these features is smaller in the gauge than in the grip, since the creep process induces strain recovery and redistribution. At 29 MPa, strain bands exhibiting an average misorientation of 1.3° are observed. The strain localization features at 21 MPa are very similar to those observed at 29 MPa and are not shown here for the sake of brevity. In essence, the KAM maps show creep-induced rearrangement and localization of strain by formation of bands, with the level of strain accumulation increasing from 21 to 29 MPa.

**Figure 11:**
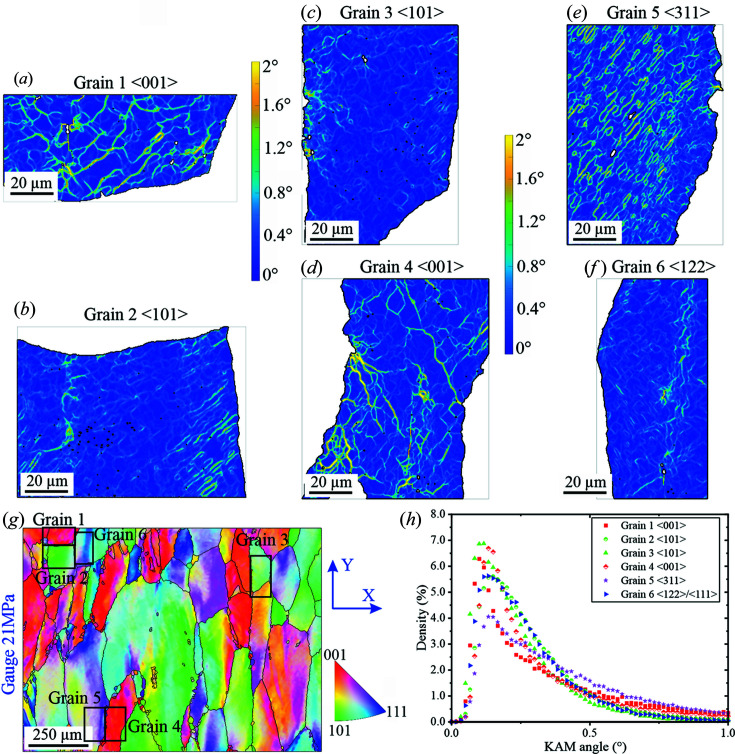
(*a*)–(*f*) EBSD–KAM maps of some individual selected grains in Al 99.8% at 21 MPa and 2.5% deformation. (*g*) EBSD orientation map indicating the location of the selected grains. The IPF colour reference triangle is for the radial direction. (*h*) A KAM density histogram of the selected grains.

As shown in Fig. 11 ▸, KAM maps and histograms of isolated grains at ×2000 magnification allow the comparison of crystal orientations of Al-99.8% at 21 MPa. Differences in the spatial arrangement of subgrains can be observed among different crystal orientations. These differences seem more pronounced between {001} and {101} grains: the misorientation value at the subgrain walls barely reaches 1.6° in {101} grains [Figs. 11 ▸(*b*) and 11 ▸(*c*)] while it reaches values higher than 2° in {001} grains. The {311} grains seem to possess overall the highest average misorientation >2° [Fig. 11 ▸(*h*)], *i.e.* similar to (001) grains [Fig. 11 ▸(*e*)]. Furthermore, {001} grains tend to form coarser subgrains than those formed in {101} and {311} grains (which are similar to one another) [Figs. 11 ▸(*a*), 11 ▸(*d*) and 11 ▸(*e*)]. The {311} grains seem to possess overall the highest average misorientation [Fig. 11 ▸(*h*)]: subgrains have similar sizes to those observed in {101} grains, and the highest misorientation value reaches >2° [*i.e.* similar to {001} grains, Fig. 11 ▸(*e*)]. Finally, the spatial arrangement of dislocations in {111} grains and the level of misorientation are observed to be quite similar to those observed in {101} grains [Fig. 11 ▸(*f*)].

Note that EBSD maps of the Al–3.85%Mg material at high magnification (×2000) do not yield any additional information to the results shown in Fig. 10 ▸ (*i.e.* the images at ×250).

## 4. Discussion

As is known, the macroscopic RS associated with the extrusion process of the alloy relaxes in the undeformed sample during the heat treatment preceding the creep tests. As suggested previously (Millán *et al.*, 2021 ▸), however, the microscopic RSs of individual grains (Type II stress), which also originate during the material’s manufacture (extrusion), are assumed to be very stable and may remain during the annealing treatment preceding the test. One possible way of relaxing these microstresses (apart from recrystallization, *i.e.* submitting the materials to extremely high temperatures, allowing the development of new, clean, grains) would be a process such as creep deformation, which modifies the internal grain misorientation. Creep modifies the misorientation in a polycrystal by the generation of dislocation structures. Deformation-induced dislocation patterns have previously been observed by TEM in Al-99.8% and Al–3.85%Mg deformed under creep (Fernández *et al.*, 2018*a* ▸).

In creep-deformed samples, the average microstrain, which is proportional to the dislocation density, decreases slightly from the undeformed state to about 2–3% creep strain. Since stress relaxation occurs by dislocation motion (Bruno *et al.*, 2004 ▸), this observation (Fig. 4 ▸) suggests a dislocation structure rearrangement (Fernández *et al.*, 2018*a* ▸; Carneiro & Simões, 2020 ▸). Under our creep conditions (tests conducted at moderate temperature and stress), only dislocation motion, *i.e.* without any contribution of recrystallization or grain boundary sliding, is considered to drive the deformation. Grain boundary sliding can be excluded in our case, since the grain size is rather large (100 µm).

The dynamics of dislocations during creep are reflected in the evolution of (intragranular) microstrains. In our case, the influence of type II stress on the peak broadening is minimized by the use of the radial strain component in the neutron diffraction experiment. In fact, both the fibre texture of the materials and the load have the same effect in all transverse directions because of the cylindrical symmetry. This allows a consistent comparison between EBSD and neutron diffraction results. According to our results, the evolution of the dislocation structure during creep deformation of Al-99.8% depends on the grain crystallographic orientation. The plastic deformation of an individual grain depends not only on its own orientation but also on the orientation and deformation state of its neighbouring grains (Al Mamun *et al.*, 2021 ▸). Despite there being important differences between the room-temperature and creep conditions, it is also found here that, as the strain progresses, the stress concentration rises close to the grain boundaries of grains unfavourably oriented to the applied stress. This is indirectly visible in the microstructures shown in Fig. 9 ▸. According to the microstrain evolution shown in Fig. 6 ▸, all grain families behave similarly except for {111}.

A reduction in the microstrain is produced during secondary creep in all the grain families investigated for 21 and 29 MPa, except for {111} at 21 MPa. This is the only difference found between the power-law (21 MPa) and power-law breakdown (29 MPa) regimes. This effect could be related to the higher dislocation density generated at 29 MPa, especially screw dislocations.

The rearrangement of dislocation structures is mainly produced at high temperature by the activation of dislocation climbing: *i.e.* dislocations move out of their slip plane, providing dislocation recombination and rearrangement into subgrain boundaries. In Al-99.8% this rearrangement reduces the microstrains with respect to the undeformed state. However, the {111} microstrain increases in the steady state at 21 MPa. This result indicates a high dislocation activity in {111} grains during creep, which can be explained by cross-slip activity. In f.c.c. crystals, dislocations can split into Shockley partials connected by a stacking fault in {111} glide planes (Kysar *et al.*, 2010 ▸). For cross slip to take place, a screw dislocation must be locally constrained before it can dissociate into intersecting {111} planes (Kysar *et al.*, 2010 ▸). This process can occur by thermal activation aided by an external stress, exactly as in the case of creep. The local stress concentration is proportional to dislocation density, which is higher at 29 MPa. This favours dislocation annihilation and rearrangement, which is reflected in microstrain reduction. The higher degree of dislocation rearrangement at 29 MPa would generate highly ‘refined’ subgrain boundaries that would enhance diffusion during creep. This would explain the power-law breakdown.

There is also another important factor related to the influence of crystallographic orientation of grains on the creep behaviour of extruded Al-99.8%. The {111} grain population is the largest (Table 1 ▸). Therefore, the strain inhomogeneity among grains is shared by two populations, {111} and the rest of the grains. This generates a stress concentration at the {111} grain boundaries that progresses with creep strain up to sample fracture.

The microstrain evolution with creep deformation in Al–3.85%Mg is (in contrast to Al-99.8%) negligible for all grain families at both 21 and 29 MPa. This weak dependence of the microstrain evolution on the applied creep stress explains the absence of power-law breakdown for Al–Mg alloys (Serrano-Munoz *et al.*, 2023 ▸). In the Al–3.85%Mg alloy, the Mg atoms anchor the dislocations, thereby limiting their climbing ability. For this reason, these alloys do not generate subgrain structures during high-temperature deformation, but rather dislocation forests (Sherby & Burke, 1968 ▸). These structures consist of a highly interconnected arrangement of dislocations that locks intragranular and intergranular microstrains to their initial value after production, as can be seen by comparing Figs. 7 ▸ and 8 ▸. Al–Mg alloys are characterized by a high deformation capacity under creep (Kysar *et al.*, 2010 ▸). This behaviour is well explained by the present results: the microstrain remains almost constant during the whole creep test (Fig. 8 ▸). The lack of microstructure evolution (dislocation forests marginally change their arrangement) is fully in line with the constancy of the microstrains throughout all creep stages, and fully explains why in Al–Mg the tertiary creep regime stretches over long timescales and does not show an exponential behaviour. In Al–Mg alloys, dislocations move along the same slip planes within each grain until they recombine/store at the grain boundary. The process repeats itself continuously, allowing the material to reach large deformations.

In Al-99.8%, instead, the microstructure evolves, as do the microstrains. At the same time, creep proceeds at much faster rates than in Al–Mg and failure occurs much earlier. This tight correlation between microstrains and dislocation structures indirectly proves the link between dislocation structures (determined by both neutron diffraction and KAM) and the creep behaviour of our materials: Al–Mg is able to accommodate large creep deformations over a long period of time, even under tertiary creep conditions, because of its microstructural stability. Conversely, Al-99.8% can only accommodate limited creep deformation because of its faster microstructure (and microstrain) evolution.

According to our analysis of the neutron diffraction peaks, the grains presenting the lowest average microstrain in Al 99.8% are {111} at 21 MPa and {111}+{200} at 29 MPa (Fig. 7 ▸). In the case of the Al–3.85%Mg alloy, the grains with the lowest average microstrain are {111}+{200} at 21 MPa and {111} at 29 MPa (Fig. 8 ▸). It seems that the modification of the global texture by the presence of Mg in solid solution reverses the sequence of microstrain content in the different families of grains. In fact, a comparison between the microstrain accumulation and the misorientation analysis performed by KAM shows that the {200} grains in Al 99.8% have a lower KAM density for low KAM values, but higher at high KAM values (Fig. 11 ▸).

The stress field generated by an individual dislocation has been well known for decades (Brailsford, 1966 ▸). However, developing a multiscale description of the stress field generated by a structure of dislocations is possibly one of the most difficult problems in materials science. In pure Al, the crystallographic orientation of a grain with respect to its neighbours determines the subgrain size (Blum, 1991 ▸). The resulting stress state of such a substructure is inversely proportional to the number of active slip systems with respect to the applied stress. The complementarity of EBSD–KAM and neutron diffraction techniques is fundamental here in establishing the correlation between crystallography, dislocation structure and the associated stress field. To the best of our knowledge, this is the first time that the influence of crystallographic orientation on creep-induced dislocation configurations in metallic materials has been investigated. This result opens the door to the development of a unified model describing the mechanical behaviour of room-temperature stationary and high-temperature dynamic deformation (creep).

## 5. Summary and conclusions

In the present investigation we have used neutron and electron backscattering diffraction to investigate the evolution of microstrain and dislocation densities (and arrangements) as a function of creep strain in Al-99.8% and Al–Mg alloy. We have also investigated the dependence of such dislocation densities on the crystallographic orientation of grains.

The following are the main conclusions of this investigation:

(i) In pure Al, the microstrains of all grains are greatly reduced during steady-state creep, regardless of the applied stress, except for {111} grains. Moreover, the dislocation density evolves as a function of creep time, and the KAM distribution (measured at 2.5% strain) of different crystal families is different.

(ii) In Al–Mg alloy, the microstrains of all grains remain unchanged during creep deformation, regardless of the applied stress. Moreover, while the dislocation density is higher than in Al-99.8%, it undergoes no significant variation upon creep deformation. Correspondingly, the KAM maps (at 2.5% strain) look homogeneous, except for a few concentration regions. The similar microstrain evolution among different grain families and the homogeneity of the dislocation arrangement explain the high creep deformation ability of this alloy.

(iii) The dislocation rearrangement of pure Al and Al–Mg alloy is strongly influenced by the grain crystallography. In particular, the microstrain associated with the grain orientation (generated in the manufacturing process, extrusion in the present case) evolves differently in pure Al and Al–Mg alloy as a function of creep strain. Such different evolution imparts (together with the microstructural evolution, *i.e.* the dislocation arrangement) the peculiar creep properties to the mater­ial.

(iv) The difference of the microstrain evolution and of the dislocation arrangement with applied stress in {111} grains is assumed to trigger the onset of the power-law breakdown regime in pure aluminium at high stresses.

## Figures and Tables

**Table 1:** Grain volume fractions *F*_v_ measured by X-ray diffraction in the axial direction for Al-99.8% and Al–3.85%Mg

Material	Texture component	*F*_v_ (%)
Al-99.8%	(111)	45
	(200)	30
	(220)	13
	(311)	12
Al–3.85%Mg	(111)	15
	(200)	50
	(220)	15
	(311)	20

**Table 2:** Strain rates at different strain levels for Al-99.8% and Al–3.85%Mg alloy

Material	Stress (MPa)	1%	2%	3%	6%
Al	21	5.2 × 10^−5^	4.4 × 10^−5^	4.8 × 10^−5^	–
Al	29	7.9 × 10^−4^	1.1 × 10^−3^	1.7 × 10^−3^	–
Al–3.85%Mg	21	3.4 × 10^−5^	–	3.4 × 10^−5^	3.5 × 10^−5^
Al–3.85%Mg	29	1.2 × 10^−5^	–	1.3 × 10^−5^	1.5 × 10^−5^
